# Enhancing pandemic surveillance and testing: a simulation modeling study utilizing german multicenter data with federated machine learning

**DOI:** 10.1007/s10729-025-09752-4

**Published:** 2026-03-14

**Authors:** Stefan Kempter, Jens O. Brunner, Frank Hanses, Christoph Spinner, Lutz T. Zabel, Christoph Römmele, Stefan Borgmann, Jörg Janne Vehreschild, Christina C. Bartenschlager

**Affiliations:** 1 Department of Technology, Management, and Economics, Technical University of Denmark, Akademivej 358, 2800 Kongens Lyngby, Denmark; 2 Health Care Operations/Health Information Management, Faculty of Business and Economics, Faculty of Medicine, University of Augsburg, Universitätsstraße 16, 86159 Augsburg, Germany; 3 Center for Excellence in Healthcare Operations Planning, Next Generation Technology, Technical University of Denmark, Fælledvej 11, 4200 Slagelse, Denmark; 4 Department for Infection Control and Infectious Diseases, University Hospital Regensburg, Franz-Josef-Strauß-Allee 11, 93053 Regensburg, Germany; 5 TUM School of Medicine and Health, Department of Clinical Medicine, Clinical Departments for Internal Medicine II, University Medical Center, Technical University of Munich, Ismaninger Str. 22, 93053 Munich, Germany; 6 Laboratory Medicine, Alb Fils Kliniken GmbH, Eichertstraße 3, 73035 Göppingen, Germany; 7 Clinic for Internal Medicine III - Gastroenterology and Infectious Diseases, University Hospital Augsburg, Stenglinstraße 2, 86156 Augsburg, Germany; 8 Infectious Diseases and Infection Control, Ingolstadt Hospital, Krumenauerstraße 25, 85049 Ingolstadt, Germany; 9 Department II of Internal Medicine, Hematology/Oncology, Goethe University, Theodor-Stern-Kai 7, 60590 Frankfurt, Germany; 10 Anaesthesiology and Operative Intensive Care, University Hospital of Augsburg, Stenglinstraße 2, 86156 Augsburg, Germany; 11 Applied Data Science in Health Care, Ohm University of Applied Sciences Nürnberg, Wassertorstraße 10, 90489 Nürnberg, Germany; 12 Faculty III – Economics, Business Informatics, Business Law, University of Siegen, Kohlbettstraße 15, 57072 Siegen, Germany

**Keywords:** Federated machine learning, hospitals, COVID-19, Multicentre data, Simulation

## Abstract

The COVID-19 pandemic has starkly exposed queryPlease check author names and affiliation if presented correctly.vulnerabilities in the management of surveillance and testing. Significant challenges associated with physical tests, i.e., PCR and antigen tests, include their high cost, resource-intensive nature, turnaround time, and sensitivity. Although the literature has underscored the potential of Machine Learning-based methods for the digital diagnosis of COVID-19, developing high-performing models crucially depends on extensive datasets exceeding the amount available in one healthcare institution. Federated Machine Learning offers a solution to that dilemma. The aim of this research is to evaluate the potential impact of Federated Learning-based digital COVID-19 diagnosis on the trajectory of a pandemic. Therefore, we design a multidimensional evaluation framework, consisting of a simulation study utilizing real-world lab parameters from multiple hospitals and a newly developed performance indicator, named Testing Evaluation for Pandemics. We find that Federated Learning can significantly support the decision-making process of diagnosing COVID-19 at the beginning of a pandemic while saving scarce resources. However, a warm-up phase is needed until constant performance similar to physical tests is reached. In addition, lab parameters have a high prediction power for the diagnosis and are well suited because of patient welfare reasons.

## Highlights

 

- We design a multidimensional evaluation framework that integrates a Federated Machine Learning simulation and an indicator consisting of economical and operational dimensions to elucidate the timing and efficacy of Federated Machine Learning during the early stages of a pandemic.
- We utilize lab parameters for diagnosing COVID-19 with Federated Machine Learning, revealing their high prediction power and suitability because of cost, resources, and patient welfare reasons.
- Federated Machine Learning can assist decision-making during the beginning of a pandemic while saving scarce resources.
- Federated Machine Learning thrives with limited data availability in a pandemic onset, enabling the use of robust local Machine Learning models later on.

## Introduction

**Fig. 1:**
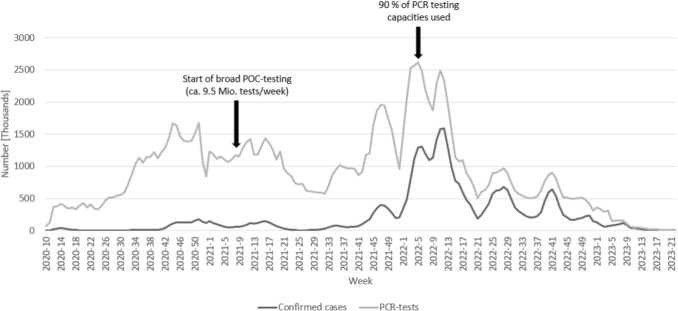
Testing during the pandemic in Germany based on data of the Robert Koch-Institute [6, 7]

The COVID-19 pandemic has starkly exposed vulnerabilities of the global supply chains and inadequacies in the decision-making processes in healthcare institutions. This was particularly evident in the management of surveillance, testing, and diagnosis of COVID-19 cases. For example, in Germany, there was a massive expansion of polymerase chain reaction (PCR) and later point of care antigen testing capacities during the pandemic (as depicted in Fig. 1). However, laboratories and testing stations frequently encountered capacity constraints [1]. Concurrently, there was an assumption of a high number of unreported cases, potentially exerting a significant impact on the pandemic’s trajectory through subsequent infections [2]. This concern is amplified by the comparatively low sensitivity of antigen tests [3] and the turnaround time of PCR tests, which can take several hours [4]. Moreover, other significant challenges associated with physical tests, i.e., PCR and antigen tests, include their high cost [4], resource-intensive nature, and lack of sustainability [5].

Throughout the pandemic, the literature has underscored the potential of Machine Learning (ML)-based methods for the digital diagnosis of COVID-19 (refer to [8] for a comprehensive review), although the inclusion and the amount of data have sparked controversy [9]. Researchers and practitioners rely heavily on extensive datasets for predictive analytics and ML applications. However, in healthcare, this poses a significant challenge due to the sensitivity of medical data (as discussed in [10, 11], and [12]) and legal regulations. Training and testing ML algorithms typically necessitate more data than any single hospital can provide [13]. Consequently, while full data access is crucial for developing high-performing models for healthcare institutions, data scientists must navigate legal, ethical, privacy, and technical constraints. Federated (Machine) Learning (FL) offers a solution to this dilemma. The relatively new research domain, which was introduced by Google in 2016 [11], ensures data privacy and governance through decentralized training and subsequent consolidation of ML models.

The purpose of this research is to show significant insights into pandemic management, elucidating the timing and efficacy of implementing digital diagnosis methods focusing on FL. Therefore, we design a multidimensional evaluation framework that consists of two parts. As a first part, we leverage a simulation study and secondary data from multiple hospitals. Notably, our study makes the first investigation into time-dependent FL-based digital COVID-19 diagnosis, encompassing varying numbers of hospitals and data distributions within the federated environment. Furthermore, we pioneer the examination of blood parameters as predictive features in this context, contrasting our approach with a baseline from a centrally hosted algorithm. As a second part, we conduct a comprehensive comparison of physical and digital testing procedures, leveraging a newly developed key performance indicator (KPI) based on multi-criteria decision making under certainty called Testing Evaluation for Pandemics (TEP). Through this analysis, we assess the value added from both operational and economical perspectives, considering factors such as performance, time efficiency, sustainability, and cost-effectiveness.

We find that FL can significantly support the decision-making process of diagnosing COVID-19 at the beginning of a pandemic while saving resources. However, the leverage of FL is dependent on the data size and label distribution of each client in a given environment. If the configuration is dominated by clients who have low data variety, we observe a decreasing performance in the technique. Additionally, adaptive optimization algorithms for FL tend to perform better than the standard algorithm but need fine-tuning. Further, we see indications that later on in a pandemic, when sufficient data is available at the participating clients, ML models restricted to local data are outperforming FL algorithms. Nevertheless, FL can be of advantage for clients who have poorer data and therefore a worse performing prediction model. Our algorithms are based on multicenter data of lab parameters, which we find have a high prediction power for the COVID-19 diagnosis. The parameters are well suited because of cost, resources, and patient welfare reasons.

Our work is structured as follows. Based on the foundations of FL, Section 2 presents related literature on FL and simulation in a pandemic. In Section 3, we introduce our multicenter dataset and the COVID-19 application from a methodological point of view. The results of the multidimensional evaluation framework consisting of the simulation and the developed indicator TEP are presented in Section 4. Section 5 discusses the results, and Section 6 concludes.

## Problem setting and related literature

This section is divided into four parts. Firstly, the theoretical concepts of FL are defined by introducing the multidimensional evaluation framework. Secondly, related literature on FL in a pandemic is introduced. Thereby, the last pandemic of COVID-19 is chosen as a primary example. Thirdly, papers on simulation studies in a pandemic are presented. Lastly, the delineation of our study and the arising contributions based on the related literature are listed.

### Multidimensional evaluation framework leveraging FL

In order to support decision-making during a pandemic onset, we develop a multidimensional evaluation framework consisting of an FL simulation and a KPI. We term the indicator TEP and apply it to the COVID-19 pandemic. For the KPI TEP, we differentiate between physical and digital testing methods and compare them according to economical and operational dimensions over time. The physical tests are the antigen and PCR tests, whereas, for the digital testing, a federated, time-dependent simulation environment is implemented. In the FL simulation, the beginning of a pandemic is recreated by utilizing a secondary dataset of patients’ blood parameters and an environment in which multiple hospitals collaborate to calculate the COVID-19 diagnosis. The collaboration of the hospitals takes place within the framework of the FL algorithm. The FL algorithm’s purpose is to learn the underlying dependencies of the data owned by multiple clients or institutions by not collecting it centrally and thus not exposing it to other collaboration partners [14]. The FL algorithm learns by applying ML techniques to a distributed setting [15]. In order to investigate the behavior of FL over the time of the pandemic outbreak, we restrict the data access of the algorithm and vary the number of participating clients, reflecting a cumulative build-up of infected patients at the start of the pandemic with a varying set of collaborating hospitals.

In general, FL can introduce two major benefits to such an environment while reducing data shortage and learning from a distributed multicenter dataset. Those benefits are enhanced data privacy and security [16]. The implementation of the FL algorithm follows either a sequential or parallel computing plan. In the remainder, a parallel FL with an aggregation server is investigated, since hospitals have relatively powerful computing resources and a reliable network connection available. A sequential computing plan, where the FL model is trained at the collaborating institutions one after another, is not applicable in this context [10].

**Fig. 2:**
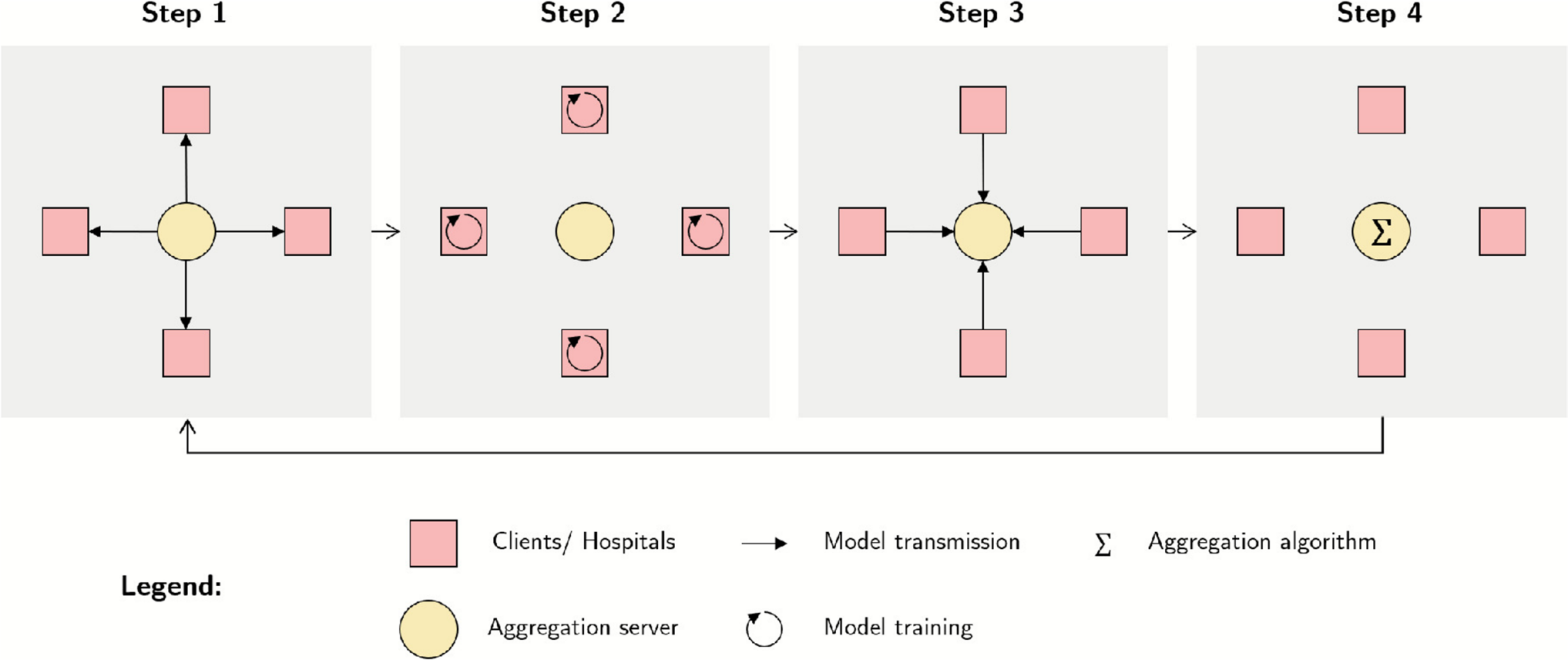
Schematic FL process with four clients and an aggregation server

If four hospitals decide to collaborate using FL with a parallel computing plan and an aggregation server, the schematic process of their collaboration can be illustrated as shown in Fig. 2. The general task of the server is to orchestrate and moderate the training of the FL model through communication with the clients. In the first step of the FL algorithm, the aggregation server sends the ML model to each of the hospitals. Then each hospital trains the received model with its locally available data. After the training, the clinics send the model back to the server. Therefore, the data stays at the local site and is not shared. In a fourth step, the server performs an aggregation algorithm when all the models from the hospitals are received. A widely established algorithm is federated averaging (FedAvg) based on [17]. Here, a weighted average of the models’ parameters is calculated. Other examples of aggregation algorithms include adaptive optimization as outlined in [18]. After the new parameters are updated, the server starts over with the first step and sends out the model again to the clinics. Steps one through four are called communication rounds and are run until the training is complete [10]. In Appendix A the applied formulas for the FL algorithm can be found.

### FL in a pandemic

The FL algorithm is widely applied and researched for the COVID-19 pandemic. As hospitals are facing multiple pandemic related challenges, such as effective and efficient testing for slowing the spread of the disease, the implementation of FL can be advantageous [16]. In this section, literature on applying the FL algorithm to the COVID-19 pandemic is presented. Thereby, the applications of FL to the pandemic can be grouped by the data type used for developing the model, as well as a category for literature reviews and conceptual frameworks.

#### Reviews

Literature reviews focus on the topic of FL or the disease in question to varying degrees. For example, [15] describe the early detection of possible diseases, including COVID-19, using FL as a tool for auxiliary diagnosis. On the contrary, [19] and [20] investigate the role of AI specifically for the COVID-19 pandemic. Both find a high potential for applying AI to the pandemic, specifically the AI algorithm FL, when facing limited data availability and ensuring data privacy. Other literature reviews target the method of FL and suggest ways to enhance data privacy. Those include [21] or [22], who are researching the combination of FL with blockchain. The article of [23] is methodically more specific and reviews literature for FL with medical images for several diseases, including COVID-19.

#### Medical images

In the second category, algorithms that are implemented on medical images are assembled. Thereby, authors use predominantly X-ray or computed tomography (CT) images. CT images are multiple X-ray images taken from different angles of the chest, to generate a three-dimensional picture for the radiologists to analyze [24]. Exemplary algorithms applied to X-ray images can be found in [25, 26] or [27] whereas CT images are used in [28, 29] or [30]. Other medical images in the form of ultrasound images are found in the studies of [31, 32], or [33]. Lastly, medical images are also used in combination with other data types. [34] are using X-ray images together with vital signs, demographic data, and lab values to predict the future oxygen requirements of a positively diagnosed COVID-19 patient. Further examples of this category include [35] and [36].

#### Multiple data types

Moreover, several authors apply the FL method to multiple data types available for a patient. [37] is gathering the patients’ historical medical information, vital signs, lab test results, and mortality outcomes from five different centers to predict the mortality rate of the positively diagnosed patients within seven days of admission. [38] and [39] use COVID-19 symptom features and cough sounds for some of their reported use cases. The use of symptom features and demographic values to predict a diagnosis is also done by [40] and [41].

### Simulation studies in a pandemic

In order to quantify the value of using FL for surveillance and testing during a pandemic outbreak, as well as to draw conclusions for future pandemics in terms of preparedness, we apply a time-dependent Monte Carlo simulation. Numerous studies have already demonstrated that simulation models are highly suitable for various healthcare applications [42–44]. It is not surprising, then, that simulations were extensively utilized during the pandemic to address a range of associated questions [45, 46]. Their value has been illustrated in several areas: [47–50], and [51] focus on COVID-19-related challenges in the intensive care unit; [52, 53], and [54] on capacity management; [55] and [56] on visitor management strategies; [57] on performing endoscopic procedures; and [58] on balancing scarce resources in hospitals. The benefits of a simulation are realized through mimicking real processes within a computer model. Additionally, simulation studies can conduct various "what-if" analyses under specific and prefixed conditions, enabling healthcare decision-makers to make informed and evidence-based decisions.

### Delineations and contributions

After grouping the relevant literature by the underlying data types and incorporating literature on simulation in a pandemic, it becomes evident that this study can contribute in three ways to existing literature. First, the usage of blood parameters or lab values for diagnosing COVID-19 in a federated way has not been researched. The studies of [34] and [37] are using lab values not to predict the diagnosis, but to predict mortality or future oxygen level of infected patients. Additionally, they combine the parameters with other features of importance, such as an X-ray image or the patient’s historic medical information. However, using blood values as features for the digital COVID-19 diagnosis comes with several benefits. Primarily, the values are determined routinely in the emergency department for arriving symptomatic patients [4]. Since the lab values are routinely taken, the parameters don’t lead to additional costs such as other diagnostic tools, e.g., PCR, antigen, X-ray, or CT images. X-ray and CT images are expensive to take and require highly technically skilled personnel. On top of that, they are only used in combination with the PCR tests to identify false negatives [24]. Furthermore, the methods are irradiating patients [8]. Second, to the best of our knowledge, the behavior of FL is not studied in a time-dependent manner with a varied set of participating clients and data distributions during the onset of a pandemic. Third, the advantages of using FL for diagnosis are not evaluated quantitatively along economical and operational dimensions, incorporating sustainability aspects, running costs, and time to other testing procedures. Consequently, the potential of FL for a pandemic is not comprehensively assessed in the existing literature.

## Methods

In this section, the methodology of the multidimensional evaluation framework is defined in two steps. Firstly, the simulation environment as well as the ML models, and the dataset are introduced. Secondly, the calculations for developing the TEP indicator are defined. All calculations are done using Python in version 3.10.11. The models are implemented with Keras and Tensorflow in version 2.15.0, whereas the federation of the models uses the Flower framework in version 1.8.0.

### Simulation environment

**Fig. 3:**
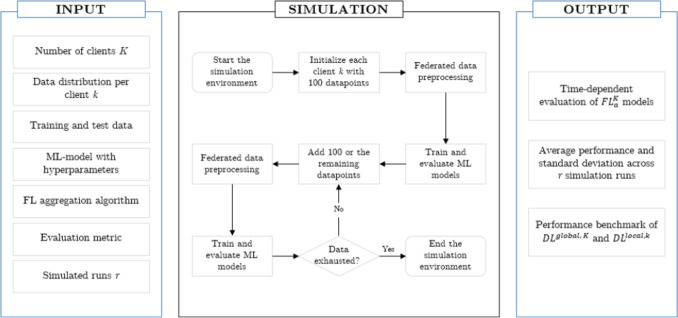
Inputs, outputs, and flowchart of the simulation environment

To evaluate the FL algorithm for the prediction of the COVID-19 diagnosis with lab parameters in a time-dependent manner, a simulation environment as described in Fig. 3 is defined. The simulation is initialized with a set of *K* clients, each assigned 100 training datapoints sampled according to a predefined data distribution. In the next simulation step, the data is preprocessed in a federated way for each client. Based on the prepared data, several ML models are defined for further analysis. Those include a Deep Learning (DL) model $DL^{local, k}$ which is run locally at each client site *k* in 1, ..., *K* and can only access the data available at that site. Further, the FL algorithm $FL^K_a$ is set up with the selected federation aggregation algorithm *a* from set *A*. Lastly, a DL model $DL^{global, K}$, which is based on the assumption of centrally aggregated data of all participating clients, is implemented. The performance of the FL algorithm can be compared to $DL^{local, k}$ and $DL^{global, K}$. Findings in the literature on applying FL to different modeling tasks within the healthcare setting have shown that the technique can achieve similar performance compared to ML models trained on all available data [25, 59]. So, the model of $DL^{global, K}$ is a hypothetical case which can be interpreted as a benchmark for the $FL^K_a$ model. After the ML training process, the models are evaluated on the static held-out dataset at the server. Thereby, the training and evaluation step for the ML models is repeated *r* times, i.e., $r = 5$, to provide information about the variability of the models to the given data excerpt. We report averages and standard deviations across the simulated runs. Subsequently, 100 datapoints are added to each of the clients *k*, and the training as well as the testing processes are rerun. The step of data introduction to the environment repeats until the training dataset is exhausted and there are no more datapoints to distribute to the clients. Therefore, we gradually increase the available observations to the ML models. Once the dataset is exhausted, the simulation of the pandemic outbreak ends. In the following, we describe the input parameters to the FL simulation environment in detail.

#### Dataset and client distribution

The dataset underlying this research work entails 3, 528 real-world patients with their respective lab parameters, age, and the COVID-19 diagnosis. The features and labels available for the prediction task are listed in Table 1 along with corresponding characteristics. Table 1 can be understood as a requirements list for the format of datapoints to participate in the FL environment. The patients are sampled from the University Hospital of Augsburg, Germany, the Alb Fils Kliniken in Göppingen, Germany, and the Lean European Open Survey on SARS-COV-2 Infected Patients (LEOSS) registry. Augsburg contributes to the research with 479 patient samples, Göppingen with 619, and LEOSS with 2,430. The latter collects data of COVID-19 patients with a strong focus on German centers. The provided data attributes were constant across the data collection sites. In summary, there are 2,581 positively tested COVID-19 patients and 947 negatively tested ones. Augsburg and Göppingen provide us with real-valued blood parameters of symptomatic patients who tested for COVID-19 in the time frame of March 2020 to June 2020. Patients are included when their PCR test confirms the diagnosis. Only in the case of the LEOSS dataset, rapid tests are an acceptable alternative. The LEOSS dataset has been collected in the period from March 2020 to November 2020. Since LEOSS considers ordinal data only, the datapoints from the hospitals are encoded to the same ranges. Approval for LEOSS was obtained by the applicable local ethics committees of all participating centers and registered at the German Clinical Trials Register (DRKS, No. S00021145). In addition, the multicenter study has been reported to the local ethics committee (20-465). The LEOSS registry was supported by the German Center for Infection Research (DZIF) and the Willy Robert Pitzer Foundation. To ensure anonymity in all steps of the analysis process, an individual LEOSS Scientific Use File (SUF) was created, which is based on the LEOSS Public Use File (PUF) principles described in [60].

For the simulation task, the datapoints are split into a test and training set by a ratio of $20\%$ test and $80\%$ training. The test set is a static, fully prepared held-out dataset at the server in the FL environment. It is used to determine and compare the performance of the ML models in the simulation. The training data is distributed to the participating clients according to the defined simulation procedures. Subsequently, the data preprocessing is done in two steps, adhering to the FL principles of data privacy and security. Firstly, empty data entries are filled for each client’s data by formulating a weighted average of the median for the respective data attribute across all clients. The weight is the number of datapoints each client is holding. Secondly, the standard scaler is used per client by calculating the mean and standard deviation for each data attribute across all participants. The summary statistics for each client are solely shared with the server. Therefore, no other client can access any intermediate results or draw conclusions on other clients’ data. The federated data preprocessing is repeated each time new data is introduced to the clients.

**Table 1:** Features in the dataset

Abbreviation	Description	Datatype	Value range
Age	patient age	integer	$[0 - 120]$
DDIM	d-dimer	integer	$[1 - 7]$
HGB	hemoglobin	integer	$[1 - 7]$
PLT	platelets	integer	$[1 - 7]$
PTT	partial thromboplastin time	integer	$[1 - 7]$
RBC	erythroblasts	integer	$[1 - 7]$
WBC	leukocytes	integer	$[1 - 7]$
cCRP	C-reactive protein	integer	$[1 - 7]$
cDBIL	serum direct bilirubin	integer	$[1 - 7]$
cGGT	serum gamma-glutamyl transferase	integer	$[1 - 7]$
cGPT	serum alanine aminotransferase	integer	$[1 - 7]$
cHST	serum urea	integer	$[1 - 7]$
cKREA	serum creatinine	integer	$[1 - 7]$
cLDH	serum lactate dehydrogenase	integer	$[1 - 7]$

For the simulation, the training datapoints are introduced in an IID and non-IID way to the participating clients. In the IID case, the datapoints are distributed independently and identically to the clients, which means that participating institutions have the same distribution of the class labels [61]. For the non-IID case, we leverage the Dirichlet distribution as described in [62] to simulate real-world label imbalance in the federated setting. Therefore, we randomly draw from $p_{l, k} \sim Dir(\alpha )$ and assign the resulting data proportions $p_{l, k}$ for each label *l* to the participating client *k*. The parameter $\alpha$ is a concentration parameter, which controls the level of label imbalance. Smaller values result in more unbalanced distributions. To have higher and lower imbalance present, we set $\alpha$ to 0.1, 0.5, 1, and 10. The concentration parameters in combination with the underlying training dataset distribution restrict the simulation to a maximum of $K = 6$. To complement the scenarios, we additionally simulate configurations with $K = 2$ and $K = 4$.

#### DL model

To run an FL algorithm, an integration of an ML model is necessary. In general, DL models are particularly well suited, because of the structure of the federation algorithm [17]. The DL model for later federation is based on the architecture proposed by [37]. [37] leverage a DL architecture in a federated learning algorithm to predict the mortality for COVID-19 patients on a comparable feature set, including lab parameters. Despite the inclusion of lab parameters in [34], the referenced DL model is not applicable in our prediction case, due to a diverging feature space including images. The selected DL model is a fully connected feedforward model with four layers, i.e., an input layer with 14 units, two hidden layers with ten and five units, and an output layer consisting of one unit. Since a binary classification problem is evaluated, we apply the binary cross-entropy loss function and optimize the loss via the ADAM algorithm. To accommodate both IID as well as non-IID distributions, we deviate from the learning rate recommendation of [63] of 0.001 for the ADAM algorithm and set it to 0.0001. The activation function in the units is the rectified linear unit (ReLU), whereas the sigmoid function is applied in the output layer. To account for smaller local data sizes and to reduce generalization error, we set the batch size to eight [64]. Lastly, we set the number of federated communication rounds to be 100 with one local training epoch in each round. To create comparability, we use 100 epochs of training for $DL^{local, k}$ and $DL^{global, K}$.

#### FL algorithm and aggregation method

The FL algorithm is set up according to Section 2.1, where a parallel compute plan and a central server is used. Since hospitals have relatively powerful resources regarding computing power and infrastructure, there is no need to select clients for a federated communication round, and each participant’s data is considered [10]. To identify performance variation in the FL method while being exposed to IID and non-IID settings, we distinguish three aggregation algorithms. Firstly, we apply federated averaging as proposed by [17]. This algorithm is considered the standard aggregation algorithm for FL settings [62]. Further, we consider FedAdam and FedYogi as introduced by [18]. Those algorithms incorporate adaptive optimization on the orchestrating server and thereby show superior performance than FedAvg in heterogeneous settings.

#### Performance metric

The outcomes are evaluated using the standard performance metric Youden’s index, which is a combination of sensitivity and specificity [65]. The equation is shown in Formula 1. Sensitivity and specificity are often used in combination and are well-suited to capture the prediction behavior of a model based on imbalanced data [66]. Sensitivity is defined as the rate that the COVID-19 diagnosis is predicted truly positive from all the observations, which are classified as COVID-19 positive. Specificity is the rate that the COVID-19 disease is predicted truly negative divided by all the observations that are negative, including the false ones [67].

1 $$\begin{aligned} {Youden's index} = {Sensitivity} + {Specificity} - 1 \end{aligned}$$

### Testing evaluation for pandemics (TEP)

The second part of the multidimensional evaluation framework is the indicator TEP. TEP is formulated based on the widely established weighted sum method (WSM) from multi-criteria decision making under certainty as described in [68]. The indicator TEP has the purpose of comparing the diagnostic testing methods for COVID-19 during the onset of a pandemic, thereby unveiling the value and behavior of FL. TEP covers the dimensions, which are motivated by Section 1, of performance in the form of the Youden’s index, running time, costs, and waste per test. The dimensions are defined in Table 2.

**Table 2:** Economical and operational dimensions of the physical and digital testing methods

Dimension	Notation	Antigen Test	PCR Test	$DL^{global, K}$	$FL^K_a$
Youden’s index	YOUDEN	0.5996	0.8198	Time-dependent	Time-dependent
Running time one test (min)	TIME	20	300	60	60
Costs of running one test (€)	COST	15	15	2.6	2.6
Waste (g)	WASTE	20	30	0	0

#### Youden’s index

Sensitivity and specificity are most used for evaluating and selecting testing methods for diagnosing COVID-19 by professionals. Additionally, they can be summarized according to Equation 1. For the antigen test, a sensitivity of 0.6170 and a specificity of 0.9826 are identified, which results in an Youden’s index of 0.5996. The PCR test has a sensitivity of 0.8614 and a specificity of 0.9584, which totals a score of 0.8198 [69]. The identified performance metrics are diverging in an acceptable range from the ones in [3] and [56]. Due to the high specificity and sensitivity of the PCR test, it is referred to as the gold standard of testing methodologies [4]. Furthermore, digital testing procedures have time-dependent accuracy. As at the beginning of a pandemic, the cases of positively and negatively diagnosed patients accumulate, the performance of the model changes with the number of inputted datapoints over time.

#### Running time and cost

The running time and the cost of running one COVID-19 diagnostic test are obtained from the University Hospital of Augsburg. The costs are identified based on an eight-hour personnel shift in 2021. In the $DL^{global, K}$ and $FL^K_a$ cases, the running time and costs reflect the time and cost until the patients’ blood parameters are available for further analysis. The algorithm is then expected to predict a diagnosis almost immediately. The PCR test has a relatively high turnaround time of 300 minutes, as the time to evaluate the test in a laboratory must be considered. On the contrary, the antigen test has the lowest running time as no intermediate steps of prerequisites in the form of a laboratory are necessary.

#### Waste

From a sustainability perspective, the digital diagnosing tools do not produce any plastic waste, whereas each PCR test leaves 30*g* and each antigen test 20*g* of waste [5]. In terms of plastic waste, the numbers deviate in a tolerable range from [70], who calculate 37*g* for the PCR test. Out of completeness and consistency, the measurements are chosen as depicted in Table 2.

#### TEP indicator

We suggest an aggregated measure, the TEP score, which combines the performance metric Youden’s index, and additional operational and economical dimensions. Since the cost, time, and waste dimensions are minimization goals, the Youden’s index is deducted from one. Further, min-max normalization is applied to eliminate units across the dimensions, which is a prerequisite for using the WSM method. The weighted sum is calculated according to Formula 2, since the performance metric is the most important to evaluate a testing procedure [69]. The weight $\gamma$ is varied between zero and one to draw conclusions on the behavior of the results.

2 $$\begin{aligned} TEP(\gamma ) = \gamma ((1 - {YOUDEN})^{norm}) + (1-\gamma )\\ (COST^{norm} + TIME^{norm} + WASTE^{norm}) \end{aligned}$$

## Results

According to the suggested multidimensional evaluation framework, the result section is divided into two parts. Firstly, the FL simulation with two, four, and six clients in an IID and non-IID setting, plus the incremental increase of datapoints, is presented. Secondly, the TEP indicator, which compares the testing methods during the onset of the COVID-19 pandemic, is shown.

### Simulation of the FL algorithm

**Fig. 4:**
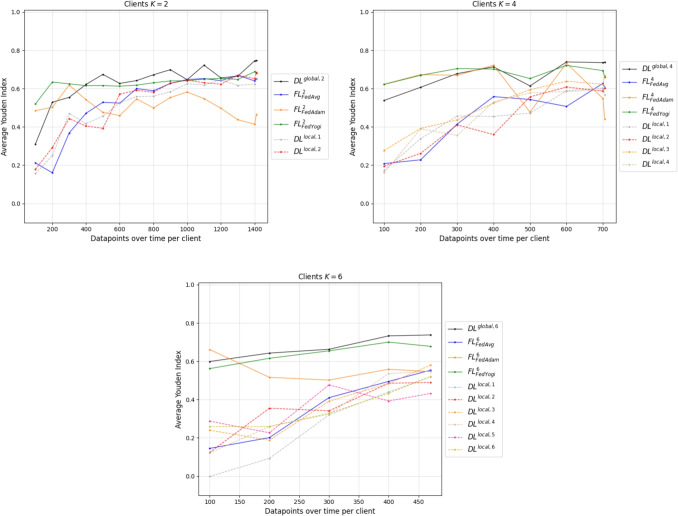
Youden’s index for the models in the IID FL environments with two (upper left), four (upper right), and six clients (lower)

In Fig. 4, the averages of the Youden’s index for an IID distribution of data between two, four, and six clients are shown. The standard deviation is in Appendix B. Note that the conclusions are based on unchanged hyperparameters throughout the simulation.

The dashed line in Fig. 4 symbolizes an average performance of the $DL^{local, k}$ models for each participating client across the five runs in the respective environment. The solid blue, green, and orange lines represent joint learning according to the FL technique with different aggregation algorithms. Furthermore, the solid black line shows $DL^{global, K}$, a hypothetical model which is trained on the centrally accumulated data of all clients. In this case, data sharing without restrictions is allowed. All results are incorporated in the graph after training the respective model.

In numbers, the simulation models of the pandemic outbreak are listed in Table 3. Thereby, all the client constitutions for the IID case are listed with their average performance and standard deviation across five runs, but the set of displayed datapoints is selected. The benchmark model $DL^{global, K}$ is added as a reference.

**Table 3:** Average Youden’s index and standard deviation for the IID simulations including $DL^{global, K}$

*K*	Datapoints per client	$FL^K_{FedAvg}$	$FL^K_{FedAdam}$	$FL^K_{FedYogi}$	$DL^{global, K}$
2	100	$0.2120 \pm 0.16$	$0.4844 \pm 0.06$	$0.5198 \pm 0.07$	$0.3104 \pm 0.12$
	400	$0.4712 \pm 0.05$	$0.5418 \pm 0.05$	$0.6155 \pm 0.04$	$0.6219 \pm 0.04$
	700	$0.5999 \pm 0.04$	$0.546 \pm 0.06$	$0.6179 \pm 0.05$	$0.6418 \pm 0.02$
	1000	$0.6474 \pm 0.03$	$0.5819 \pm 0.04$	$0.6413 \pm 0.03$	$0.6465 \pm 0.02$
	1300	$0.6691 \pm 0.02$	$0.4374 \pm 0.1$	$0.6479 \pm 0.07$	$0.6651 \pm 0.05$
	1410	$0.6525 \pm 0.02$	$0.4649 \pm 0.07$	$0.6799 \pm 0.04$	$0.7471 \pm 0.01$
4	100	$0.2085 \pm 0.11$	$0.6228 \pm 0.05$	$0.6219 \pm 0.02$	$0.5383 \pm 0.05$
	300	$0.4142 \pm 0.17$	$0.6696 \pm 0.04$	$0.7043 \pm 0.04$	$0.6781 \pm 0.02$
	500	$0.5432 \pm 0.05$	$0.4773 \pm 0.24$	$0.6526 \pm 0.05$	$0.6138 \pm 0.04$
	705	$0.6045 \pm 0.04$	$0.4408 \pm 0.1$	$0.6592 \pm 0.03$	$0.7372 \pm 0.02$
6	100	$0.1452 \pm 0.2$	$0.6612 \pm 0.03$	$0.5617 \pm 0.21$	$0.5988 \pm 0.09$
	200	$0.2014 \pm 0.2$	$0.5163 \pm 0.06$	$0.6156 \pm 0.04$	$0.6427 \pm 0.01$
	300	$0.41 \pm 0.17$	$0.5018 \pm 0.06$	$0.6545 \pm 0.03$	$0.6627 \pm 0.03$
	400	$0.4949 \pm 0.12$	$0.5584 \pm 0.03$	$0.7001 \pm 0.01$	$0.7326 \pm 0.02$
	470	$0.5532 \pm 0.07$	$0.5462 \pm 0.07$	$0.678 \pm 0.05$	$0.7375 \pm 0.01$

With full data access in the IID environment, which represents the end of the simulation, the $FL^K_a$ methods achieve comparable but lower results to the baseline performance of the $DL^{global, K}$ model. Further, the aggregation algorithm of FedYogi yields the highest average performance of 0.6799 in a configuration with two clients, whereas FedAdam leads to the lowest performance of 0.4408 in a configuration with four clients and full data access. The FedAvg aggregation algorithm lies in between. The average performance of the FedAvg algorithm is decreasing with an increase in the number of participating clients. This behavior could stem from the information loss of the weighted average method, due to including an increasing number of addends with an increasing number of clients. Additionally, the decrease in performance of the FedAdam algorithm during the datapoint increase in the simulation stands out. In each configuration environment, the models start with a better average performance than they end the simulation with.

When comparing the $FL^K_a$ to the locally trained models $DL^{local, k}$ in Fig. 4, the FL methods mostly outperform the DL models on the local clients. Here, the increase in datapoints per client increases the performance on the test set. However, in the IID case, some clients achieve comparable performance to some FL methods. For instance, in the $K=6$ environment, the clients perform similarly to the FedAvg method. When the threshold of 300 datapoints per client is exceeded, some of the clients reach the performance of the FedAdam algorithm. In an environment with two clients, the locally trained models come close to $DL^{global, 2}$, indicating that with sufficiently available data, the clients can achieve the benchmark performance. It is noteworthy that in this case, the FedAdam algorithm performs poorer than the local clients.

**Fig. 5:**
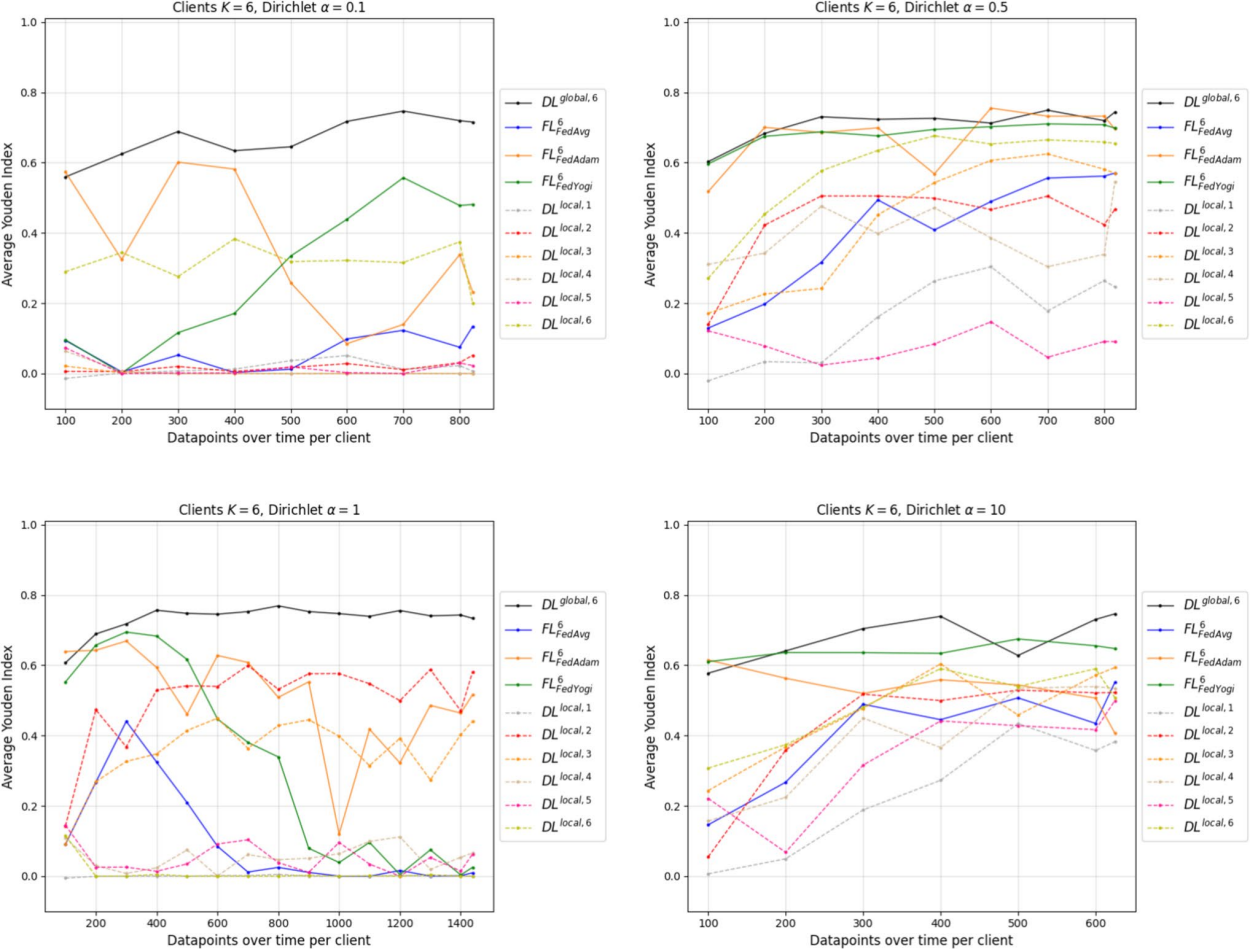
Youden’s index for the models in the non-IID FL environments with six clients

To structurally simulate a non-IID setting, we vary the Dirichlet $\alpha$ for a given client composition. The resulting client sizes and label distributions are in Appendix C. The average performance across the simulation is displayed for six clients in Fig. 5, whereas their respective standard deviation can be found in Appendix D. The graphs of the average metrics for two and four clients are in Appendix E. We further summarize the performance information in Table 4 for full data access. This represents the end of the simulation. Note that in the non-IID setting, the number of datapoints per client increases unevenly due to variations in data availability across clients. While one client may continue to add more datapoints in each iteration, another may already have reached its maximum capacity and remain static.

**Table 4:** Average Youden’s index and standard deviation for the non-IID simulations including $DL^{global, K}$ with full data access

*K*	Dirichlet $\alpha$	$FL^K_{FedAvg}$	$FL^K_{FedAdam}$	$FL^K_{FedYogi}$	$DL^{global, K}$
2	0.1	$0.6437 \pm 0.02$	$0.6537 \pm 0.07$	$0.6575 \pm 0.03$	$0.7487 \pm 0.01$
	0.5	$0.6401 \pm 0.03$	$0.6823 \pm 0.04$	$0.679 \pm 0.03$	$0.7311 \pm 0.03$
	1	$0.7055 \pm 0.01$	$0.7464 \pm 0.01$	$0.7293 \pm 0.02$	$0.7463 \pm 0.01$
	10	$0.6779 \pm 0.01$	$0.4753 \pm 0.06$	$0.6314 \pm 0.03$	$0.7169 \pm 0.02$
4	0.1	$0.7335 \pm 0.04$	$0.7432 \pm 0.01$	$0.7577 \pm 0.02$	$0.7514 \pm 0.02$
	0.5	$0.5809 \pm 0.03$	$0.6894 \pm 0.07$	$0.6216 \pm 0.03$	$0.7353 \pm 0.02$
	1	$0.332 \pm 0.17$	$0.5391 \pm 0.04$	$0.5156 \pm 0.03$	$0.7443 \pm 0.01$
	10	$0.6385 \pm 0.03$	$0.4788 \pm 0.08$	$0.6791 \pm 0.02$	$0.7465 \pm 0.02$
6	0.1	$0.1335 \pm 0.09$	$0.232 \pm 0.3$	$0.4808 \pm 0.07$	$0.7154 \pm 0.01$
	0.5	$0.5701 \pm 0.05$	$0.6957 \pm 0.05$	$0.6979 \pm 0.02$	$0.7438 \pm 0.02$
	1	$0.0095 \pm 0.02$	$0.5164 \pm 0.09$	$0.0252 \pm 0.03$	$0.7336 \pm 0.02$
	10	$0.5518 \pm 0.08$	$0.4067 \pm 0.21$	$0.6475 \pm 0.02$	$0.7462 \pm 0.01$

Table 4 underlines the applicability of FL in the non-IID setting. However, differentiations according to the skewness of the IID distribution must be made. Whereas for two clients, the overall performance of the $FL^K_a$ algorithms comes close to the benchmark, weaknesses of the approach appear with $K=4$ and $\alpha =1$, $K=6$ and $\alpha =0.1$ or $\alpha =1.0$. Those environments are heavily dominated by clients holding data from the positive label class, which reflects the majority class in the dataset. For instance, with $K=6$ and $\alpha =1$, a decrease in performance of the $FL^K_a$ in Fig. 5 can be obtained, which is starting at around 400 datapoints. This configuration environment is dominated by one client holding 1,439 datapoints with a ratio of positives being $99.37\%$. In reality, such label distributions could be achieved through integrating registries, e.g., LEOSS, which hold mostly positive data entries. The next bigger client in the environment has 351 datapoints. Therefore, from the iteration of 400 datapoints onwards, additional datapoints are only introduced by the largest client, which happens to be almost only positive datapoints. This results in skewing the model to the positive label class and reducing the Youden’s index towards zero, whereas $DL^{global, 6}$ stays constant.

Building on the IID case, Fig. 5 shows that FL can alleviate the problem of clients’ fluctuating prediction performance and higher variance. In a configuration environment of $K=6$ and $\alpha =0.5$, the average Youden’s index for the lowest performing client is at $0.044 \pm 0.05$ and at $0.6346 \pm 0.02$ for the highest performing client for 400 datapoints. FedAdam achieves $0.6989 \pm 0.04$, FedYogi $0.6762 \pm 0.02$, and FedAvg $0.4938 \pm 0.16$ in the same environment. Therefore, all local clients have an information gain by using FedAdam or FedYogi. The information gain is greatest for the lowest performing clients, where all federated approaches are advantageous. Those clients are profiting from the information held by other participating clients and can thus increase their prediction quality by participating in the federated environment.

### Application for the indicator TEP

**Fig. 6:**
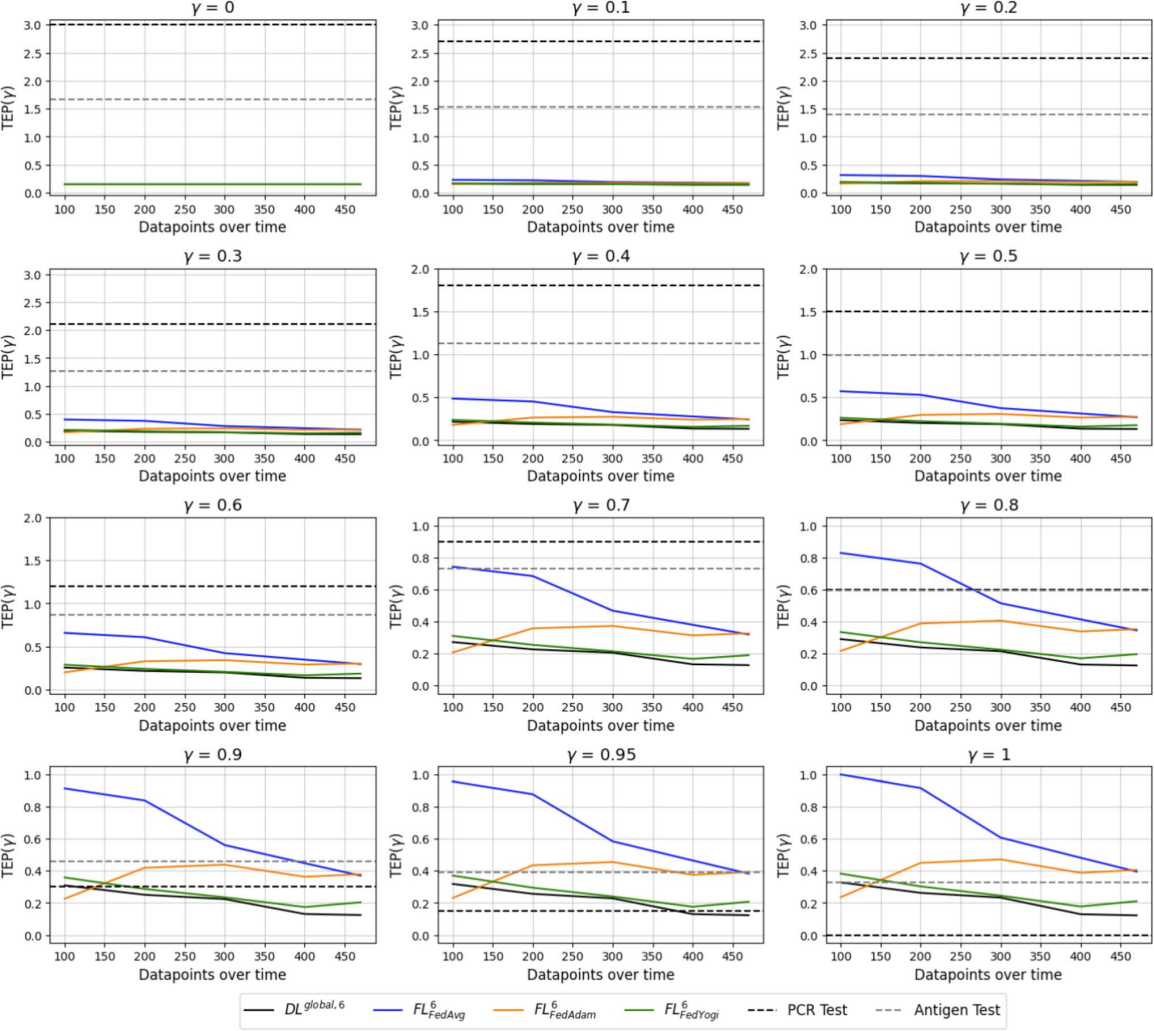
Varying weights for TEP in an IID simulation environment of six clients

**Fig. 7:**
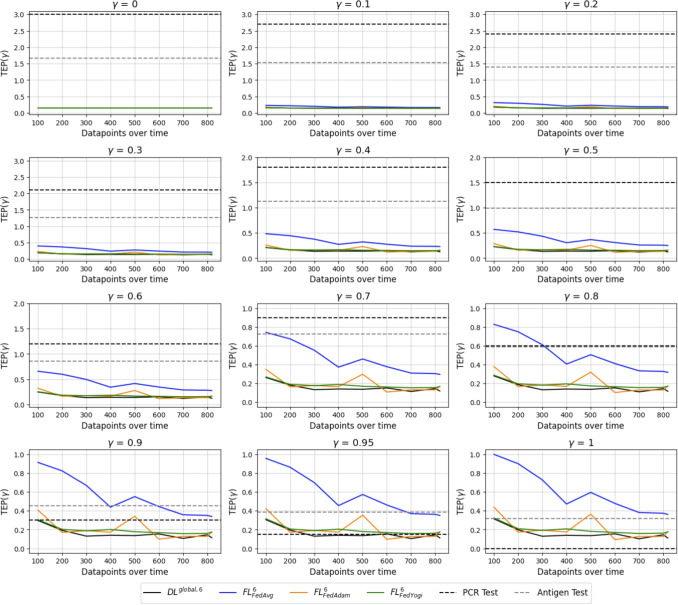
Varying weights for TEP in a non-IID simulation environment of six clients and $\alpha =0.5$

In this section, TEP is presented for an FL environment with six clients having an IID and a non-IID data distribution with Dirichlet parameter of $\alpha =0.5$. When applying Formula 2 with a varying weight $\gamma$ for the WSM, we obtain Fig. 6 and Fig. 7. Note the difference in the y-axis as the weight increases.

As all the dimensions are to be minimized, the lowest TEP values achieved are best. If the Youden’s index is not weighted, a constant line for each testing approach across time is obtained. Incorporating only costs, turnaround time, and plastic waste, the PCR test is the worst method as time progresses. Antigen tests are the second worst as they still produce plastic waste and have, compared to digital tests, relatively high running costs for one test. The digital diagnosing methods have a moderate running time since it takes some time to get the blood values for each patient. However, the algorithm doesn’t produce any plastic waste and has small costs. If the weight on the performance metric is increased, the physical tests become comparable to the digital ones.

A deviating behavior in the IID and non-IID case from the previously described ranking is obtained when the weight $\gamma$ on the Youden’s index is increased to 0.7 and beyond. Here we see a consistent ranking across the environments of FedAvg being the worst of the three aggregation algorithms. FedAdam performs better in the non-IID environment than in the IID. With a higher weight of $\gamma$, the algorithm beats the antigen test and can challenge the PCR test up to a weight of $\gamma = 0.95$. The algorithm of FedYogi performs close to the benchmark of $DL^{global, 6}$ in the IID and non-IID environments, indicating a superiority of the adaptive optimization algorithms on the server side. If the Youden’s index gets the highest weight of one, the PCR test outperforms the FL method. Nevertheless, PCR tests consume high resources in terms of cost, time, and waste, which need to be considered and incorporated into the decision-making process to not run into capacity constraints. The capacity of the PCR is not restricted in the simulation runs.

Lastly, the $DL^{global, 6}$ model on a centrally hosted dataset achieves the highest performance of all ML models. This performance challenges the PCR test in all weighted aggregations if the suggested dimensions are included with a weight different than zero in $TEP(\gamma )$. Due to the constant performance in the simulation of the PCR test, the PCR test might be beneficial at the beginning of the pandemic, but is outperformed once the digital methods have enough data available.

**Fig. 8:**
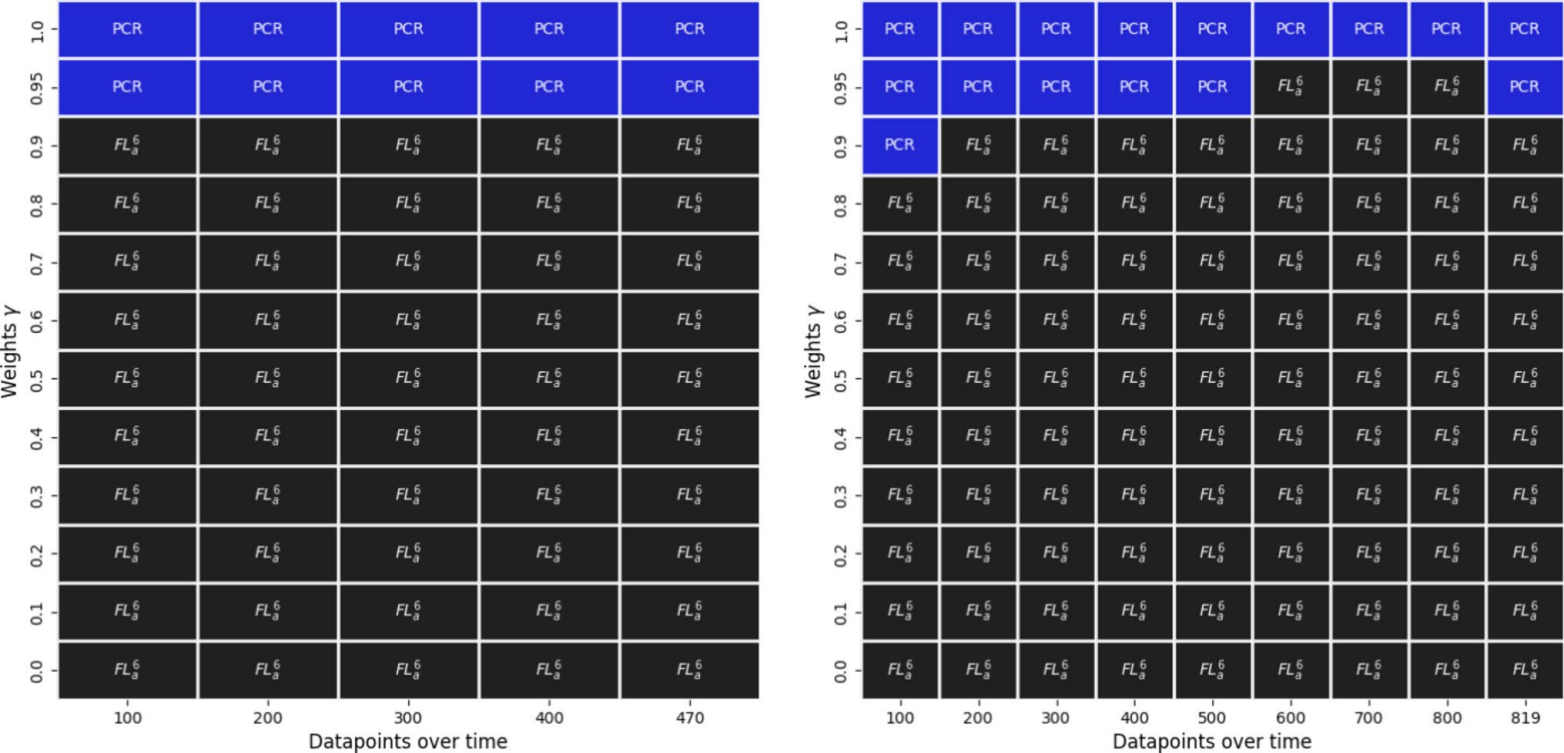
Best performing testing method across time and weights with six clients in the IID (left) and non-IID (right) environment

$TEP(\gamma )$ allows us to select the best performing testing method for the COVID-19 diagnosis across time and weight. This is visualized in Fig. 8 for the IID and non-IID environment. The best performing testing approaches to diagnose COVID-19 are the $FL^{6}_a$ model and the PCR test. The antigen test is constantly outperformed across all weight and datapoint combinations. The PCR approach becomes increasingly favorable, as the weight $\gamma$ on the Youden’s index gets larger. Especially with a $\gamma =1$, the PCR test is the best method across the whole simulated course of the pandemic. With a lower weight, the advantages of the PCR test are reduced to the immediate beginning of a pandemic, indicating the high applicability of the FL method in this context.

## Discussion

With the results of the multidimensional evaluation framework, we show the potential and drawbacks of three different FL aggregation methods and their comparison to COVID-19 testing methods such as antigen or PCR testing during the onset of a pandemic. It becomes evident that FL has advantages when sharing data on a central server is not possible. This is shown when comparing the FL approach to the benchmark $DL^{global, K}$ algorithm. Additionally, FL can soften fluctuations in locally trained models and bring information gains to participating clients, where local models aren’t able to learn as well from the available data. Here, depending on the aggregation algorithm, FL generally outperforms local models.

Nevertheless, we identified three main challenges for FL through our time-dependent simulation. Firstly, through the structured variation of the data imbalance at the clients, it was possible to display weaknesses of the FL approach. Not only the FedAvg aggregation algorithm, but also FedAdam and FedYogi were underperforming and leading to high performance gaps to the benchmark model, e.g., when $K=6$ and $\alpha =0.1$. In those environments, clients with one label class were dominant. Secondly, especially in non-IID settings, FedAvg gets outperformed by adaptive optimization algorithms. Those methods result in better performance, but might also need more fine-tuning. This task can be especially hard to perform, since data access in an FL environment is restricted. On the other hand, FedAvg is an intuitive method to understand where no further fine-tuning of the aggregation algorithm is needed. Thirdly, increasing the number of datapoints at local clients did not consistently yield performance gains. While this was true under IID conditions, in non-IID settings, we have shown instances where additional data led to stagnating or decreasing performance.

Next, our developed indicator TEP quantifies the advantages and disadvantages of physical testing devices compared to digital procedures. Based on different weights of the performance metric Youden’s index, TEP calculates the most applicable testing procedure. The physical testing devices have drawbacks such as higher plastic waste and higher cost. Only the turnaround time for antigen tests is lower than for digital methods. The FL methods can challenge the PCR and antigen test in certain environments, but fail to do so consistently across a variety of configurations. Here, not only the data distributions at the local clients but also the current state of the pandemic outbreak and the dependency on other economical and operational dimensions are relevant. Lastly, it can be obtained that at the beginning of a pandemic, the physical tests deliver a constant performance value, whereas the digital ones need more time until convergence. This learning can only happen if sufficient data is available. The result of which testing method to choose in a pandemic is dependent on how important the Youden’s score is in contrast to the other dimensions.

A comparison between FL and other diagnosing methods of COVID-19 based on different dimensions was not found in the literature and is, therefore, closing a research gap. Although diagnosing COVID-19 with FL is well researched, blood parameters for FL come in short and their potential is not fully explored. FL based on blood parameters does achieve a lower but comparable performance in terms of the Youden’s index than the gold standard of COVID-19 testing, which are PCR tests. Therefore, digital testing methods can add a suitable alternative to the already established physical ones. Even if data sharing is problematic, FL offers a suitable alternative.

The research at hand comes with two primary limitations. First, the simulation underlies assumptions. One of them is that we were able to structurally recreate label imbalance at local clients, but cannot cover all the eventualities of a real-world pandemic outbreak in the simulation model. The data heterogeneity, as well as the number of simulated clients, is bounded by the label distribution of the data available to this study. Second, Youden’s index combines sensitivity and specificity and reduces a very high specificity if the sensitivity is much lower. Depending on the progression and severity of the next pandemic, the two metrics might be differently weighted. However, [69] show that for either wrong prediction, which results from a lower sensitivity or specificity in diagnosing COVID-19, there are costs and risks associated with it.

## Conclusion

In the work at hand, we suggest a multidimensional evaluation framework with the goal of comparing the timing and efficacy of FL to physical testing procedures during a pandemic onset. Thereby, we study the behavior of FL for supporting the decision-making process in a pandemic and show opportunities as well as limitations of the method. Additionally, we are setting different diagnosing approaches in contrast to each other by quantitatively comparing them along operational and economical dimensions with our suggested indicator TEP.

With our approach, we pioneer the use of FL for diagnosing COVID-19 with lab parameters and find a high prediction power. Additionally, the values bring benefits in aspects of costs, resources, and patient welfare. Through our simulation, we found that FL can support the decision-making process at the beginning of a pandemic while saving resources. Furthermore, FL is dependent on data availability and the label distributions at the participating clients. If clients are dominating an FL environment and have a low data variety, the FL algorithm can reach its limitations. On the other hand, the FL algorithms are helpful for clients, where ML models hardly learn. In summary, we show how FL is dependent on data availability, data characteristics, and datapoints held by each client. Lastly, our newly designed indicator TEP gives an indication that physical testing methods can reach their limits. Even the gold standard of PCR can be challenged and has several drawbacks.

The retrospective perspective on the decision-making process in the COVID-19 pandemic gives a primary example of learning for future pandemic waves. Using further simulations and measurements for applying FL to improve decision quality can show the necessity of digital testing methods. Additionally, the benefits of FL can be realized in upcoming pandemics. Especially when taking the multidimensionality, including costs, running time, capacity limitations, and waste into consideration, for the future development of fast and reliable testing methodologies. As a first step in the direction of preparing for a pandemic with FL, the needed collaborations, as well as the legal and ethical framework, could be designed and set up between hospitals. This step is a prerequisite and can already take place before the next pandemic breaks out.

## Figures and Tables

**Fig. 9:**
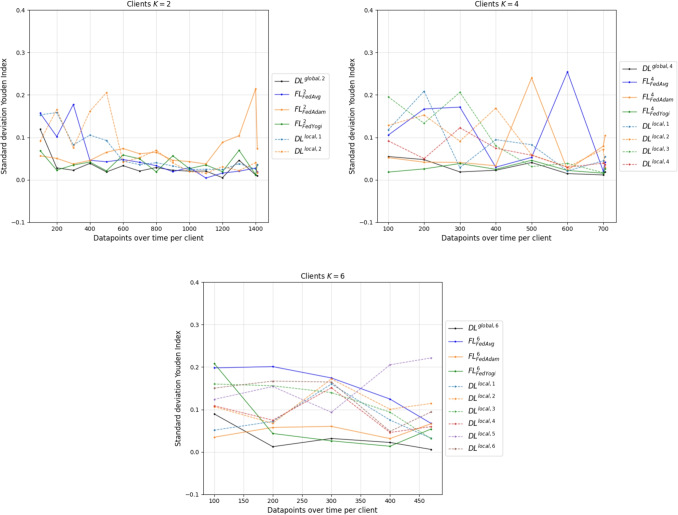
Standard deviation of Youden’s index for the models in the IID FL environments with two, four, and six clients

**Fig. 10:**
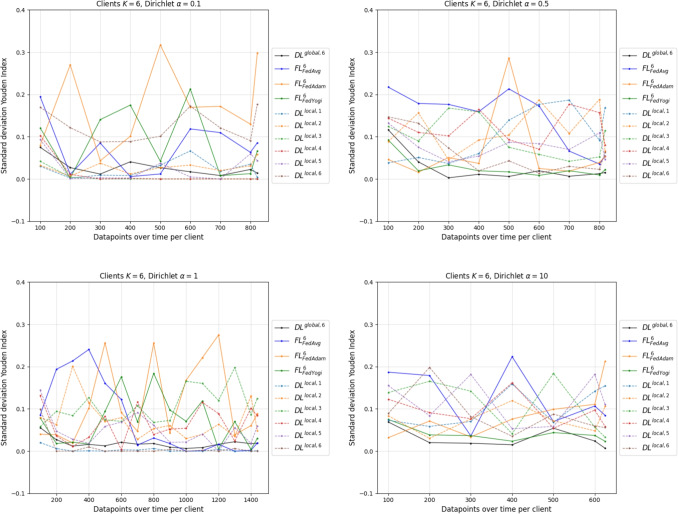
Standard deviation of Youden’s index for the models in the non-IID FL environments with six clients

**Fig. 11:**
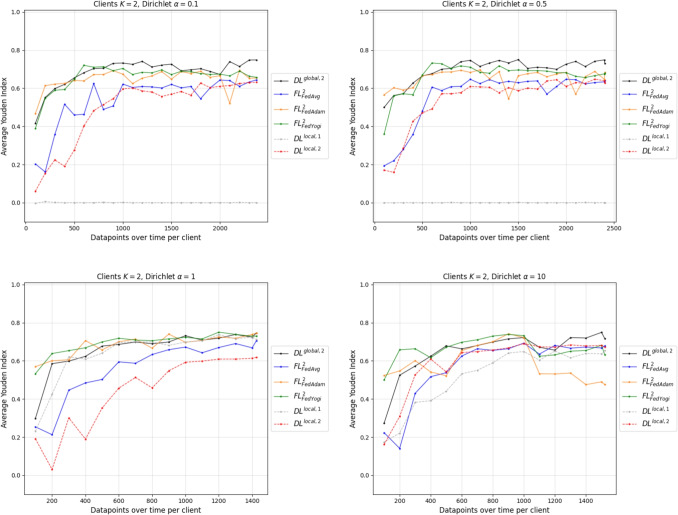
Average of Youden’s index for the models in the non-IID FL environments with two clients

**Fig. 12:**
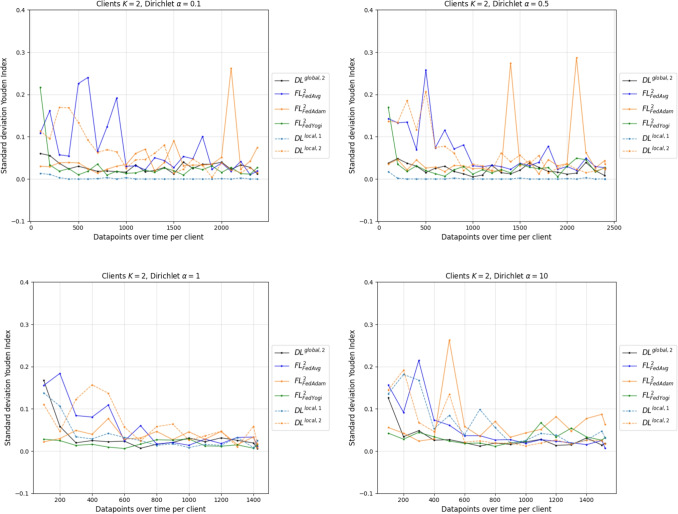
Standard deviation of Youden’s index for the models in the non-IID FL environments with two clients

**Fig. 13:**
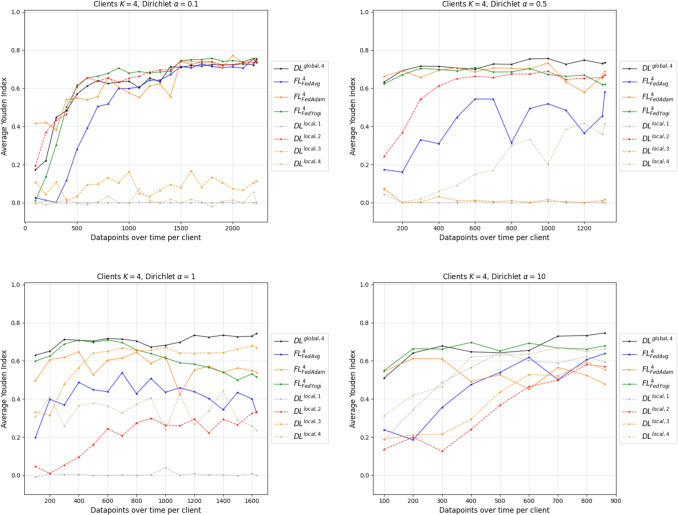
Average of Youden’s index for the models in the non-IID FL environments with four clients

**Fig. 14:**
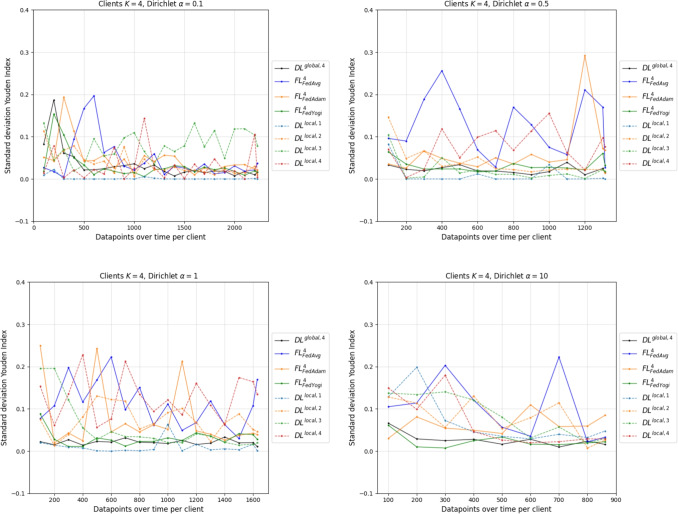
Standard deviation of Youden’s index for the models in the non-IID FL environments with four clients

## Appendix A FL theory

While differences exist in the sequence of training the FL model, all the approaches solve the same optimization problem and minimize a loss function $L(\theta )$ across all clients with respect to the real numbered model parameters $\theta \in \mathbb {R}^2$. The target function of the optimization problem is given as follows.

A1 $$\begin{aligned} \min _{\theta \in \mathbb {R}^2} L(\theta ) \end{aligned}$$

The loss $L(\theta )$ is defined by the average loss calculated from the datapoint samples $i \in {1,..., n}$.

A2 $$\begin{aligned} L(\theta ) = \frac{1}{n} \sum _{i = 1}^{n} L_i(\theta ) \end{aligned}$$

If *K* different clients or institutions, i.e., hospitals contribute to the FL model with $n_k$ data samples each, the loss formula across all clients can be rewritten.

A3 $$\begin{aligned} L(\theta ) = \sum _{k = 1}^{K} \frac{n_k}{n} L_k(\theta ) \end{aligned}$$

A4 $$\begin{aligned} L_k(\theta ) = \frac{1}{n_k} \sum _{i \in P_k} L_i(\theta ) \end{aligned}$$

Thereby, $P_k$ defines the set of samples held by client *k*. The structure of the predefined formula enables an integration of FL with several ML models. In particular, Deep Learning (DL) models are well suited for this approach [17].

Parallel FL techniques assume an aggregation proceeding to combine all the parameters computed locally at the clients’ side. Here a server can be used to update the parameters according to the individual results calculated, for example, based on the federated averaging algorithm initially proposed by [17]. The algorithm is considered a standard aggregation procedure for FL [15]. [17] assume an application of the same ML model per client and build their algorithm upon the stochastic gradient descent optimization technique. In every federated round *t*, all clients calculate one step of the stochastic gradient descent algorithm for an update of the model parameters $\theta$. Afterwards, the results are sent to the server which aggregates the parameters by computing a weighted average. The consolidated parameters are then sent back to the clients for further training or the next federated round. The update function is formulated in Formula A5 for all *k*.

A5 $$\begin{aligned} \theta _{t + 1}^{k} = \theta _t - \eta g_k \end{aligned}$$

As with conventional gradient descent algorithms, a learning rate $\eta$ is assumed. The variable $g_k = \nabla L_k(\theta _t)$ denotes the stochastic gradient computed locally based on the directional derivatives included in $\nabla$. For the federated round $(t + 1)$ the server averages the parameters according to the number of datapoints the respective client *k* is holding.

A6 $$\begin{aligned} \theta _{t + 1} = \sum _{k = 1}^{K} \frac{n_k}{n} \theta _{t + 1}^{k} \end{aligned}$$

## Appendix C Client sizes and label distributions of non-IID simulation environment

**Table 5:** Client sizes and label distribution of non-IID simulation environment with $K=2$

Dirichlet $\alpha$	Client *k*	Total datapoints	COVID-19 positives	COVID-19 negatives	Ratio positives [%]
0.1	1	448	0	448	0.00
	2	2374	2065	309	86.98
0.5	1	418	0	418	0.00
	2	2404	2065	339	85.9
1	1	1425	894	531	62.74
	2	1397	1171	226	83.82
10	1	1521	1150	371	75.61
	2	1301	915	386	70.33

**Table 6:** Client sizes and label distribution of non-IID simulation environment with $K=4$

Dirichlet $\alpha$	Client *k*	Total datapoints	COVID-19 positives	COVID-19 negatives	Ratio positives [%]
0.1	1	277	277	0	100.00
	2	2228	1497	731	67.19
	3	206	181	25	87.86
	4	111	110	1	99.10
0.5	1	363	0	363	0.00
	2	861	566	295	65.74
	3	285	276	9	96.84
	4	1313	1223	90	93.15
1	1	287	4	283	1.39
	2	1630	1510	120	92.64
	3	744	492	252	66.13
	4	161	59	102	36.65
10	1	546	382	164	69.96
	2	862	692	170	80.28
	3	792	599	193	75.63
	4	622	392	230	63.02

**Table 7:** Client sizes and label distribution of non-IID simulation environment with $K=6$

Dirichlet $\alpha$	Client *k*	Total datapoints	COVID-19 positives	COVID-19 negatives	Ratio positives [%]
0.1	1	572	552	20	96.50
	2	823	791	32	96.11
	3	496	496	0	100.00
	4	639	0	639	0.00
	5	178	178	0	100.00
	6	114	48	66	42.11
0.5	1	469	405	64	86.35
	2	313	210	103	67.09
	3	819	655	164	79.98
	4	188	76	112	40.43
	5	465	430	35	92.47
	6	568	289	279	50.88
1	1	269	0	269	0.00
	2	351	237	114	67.52
	3	317	77	240	24.29
	4	106	10	96	9.43
	5	340	311	29	91.47
	6	1439	1430	9	99.37
10	1	625	513	112	82.08
	2	348	232	116	66.67
	3	373	242	131	64.88
	4	594	437	157	73.57
	5	514	405	109	78.79
	6	368	236	132	64.13
